# Comparison of One versus Two Fecal Immunochemical Tests in the Detection of Colorectal Neoplasia in a Population-Based Colorectal Cancer Screening Program

**DOI:** 10.1155/2016/5914048

**Published:** 2016-12-01

**Authors:** Sarvenaz Moosavi, Robert Enns, Laura Gentile, Lovedeep Gondara, Colleen McGahan, Jennifer Telford

**Affiliations:** ^1^Division of Gastroenterology & Hepatology, Department of Medicine, University of British Columbia, Vancouver, BC, Canada; ^2^British Columbia Cancer Agency, Colon Screening Program, Hereditary Cancer Program, University of British Columbia, Vancouver, BC, Canada

## Abstract

*Objective.* To determine the positive predictive value (PPV) of two versus one abnormal FIT in the detection of colorectal neoplasia in a Canadian population.* Methods.* Three communities enrolled in a colorectal cancer (CRC) screening pilot program from 01/2009 to 04/2013 using 2 FITs. Data collected included demographics, colonoscopy, pathology, and FIT results. Participants completed both FITs and had one positive FIT and colonoscopy. PPV of one versus two abnormal FITs was calculated using a weighted-generalized score statistic. A two-sided 5% significance level was used.* Results.* 1576 of 17,031 average-risk participants, 50–75 years old, had a positive FIT. Colonoscopy revealed 58 (3.7%) cancers, 419 (31.6%) high-risk polyps, and 374 (23.7%) low-risk polyps as the most significant lesion. PPV of one versus two positive FITs for cancer, high-risk polyps, and any neoplasia were 1% versus 8%, 20% versus 40%, and 48% versus 67%, respectively (*p* value < 0.0001). When the first FIT was negative, the second positive FIT detected 7 CRCs and 98 high-risk polyps.* Conclusions.* PPV of two positive FITs is superior to one positive FIT for CRC and high-risk polyps. The added value of the second FIT was 12% of total CRCs and 23% of total high-risk polyps.

## 1. Introduction

Colorectal cancer (CRC) is the second leading cause of cancer death in North America [1]. Colon screening has been shown to decrease CRC mortality and incidence [2, 3]. Several strategies incorporating different screening tests and various intervals have been recommended, including the fecal immunochemical test (FIT), performed either annually or biennially [4–10].

The FIT is superior to the guaiac-fecal occult blood test (gFOBT) in the detection of CRC and advanced neoplasia [11]. In addition, participation with FIT is higher in randomized trials, compared to gFOBT, flexible sigmoidoscopy [12], and colonoscopy [13]. FIT also has been shown as a cost-effective strategy in screening for CRC at reasonable levels of willingness to pay [14, 15]. The World Health Organization recommends FIT as the test of choice for population-based screening programs [16].

The performance of FIT in the detection of colorectal neoplasia may be affected by several variables, including the number of specimens collected per screening round. In theory, CRCs bleed intermittently; however, there are limited and contradictory data available assessing the effect of the number of FIT specimens per screening round [17, 18]. Additionally, increased resources are required for multiple samples, and the cost-effectiveness of this approach is not clear.

The objective of this study is to compare the positive predictive value (PPV) of one versus two positive FITs and to estimate the value added by a second FIT specimen per screening round for the detection of colorectal neoplasia in a population-based screening program.

## 2. Methods

Eligible subjects were asymptomatic, average-risk men and women 50 to 75 years of age participating in a CRC screening pilot program, Colon Check. From January 2009 to April 2013, three communities in the province of British Columbia (BC) were invited to complete two FIT kits and undergo a follow-up colonoscopy, if either FIT was positive.

The current study included all Colon Check participants who completed both FITs, had at least one positive FIT, and underwent colonoscopy. Data was collected prospectively for the program and stored in a central database. Data reviewed for the purpose of this study includes participant demographics, FIT results, colonoscopy results, and pathology results.

Potential participants were registered through a central call center and were excluded for any of the following reasons: active rectal bleeding; personal history of CRC; personal history of Crohn's disease or ulcerative colitis; or a colonoscopy or a flexible sigmoidoscopy in the previous 5 years.

Participants received two FIT kits in the mail and were instructed to take one sample each from two consecutive bowel movements. The kits were transported to a central laboratory for analysis. A semiautomated quantitative FIT, OC-Auto Micro 80® (Polymedco, New York, USA and Somagen, Canada) was used. The FIT was considered positive, if either test was ≥100 ng/mL buffer (20 microgram hemoglobin/grams feces). If the FIT was positive, then colonoscopy was recommended.

The colonoscopies were performed by community physicians, who completed a standard reporting form documenting colonoscopy quality indicators, polyp morphology, and type of resection. Tissue specimens were assessed by BC Cancer Agency pathologists and reported in a synoptic format.

Pathology was classified based upon the most significant lesion. High-risk polyps were defined as adenomas ≥10 mm in size and ≥25% villous features, adenomas with high-grade dysplasia, sessile serrated adenomas/polyps, traditional serrated adenomas, and multiple (≥3) tubular adenomas. Low-risk polyps were one or two tubular adenomas <10 mm in size.

The PPV for cancer or advanced neoplasia at colonoscopy was compared in patients with one and two positive FITs using a weighted-generalized score statistic proposed by Kosinski [19] with a *p* value less than 0.05 considered significant.

The Human Ethics Board at the British Columbia Cancer Agency reviewed and approved this study.

## 3. Results

**Figure 1:**
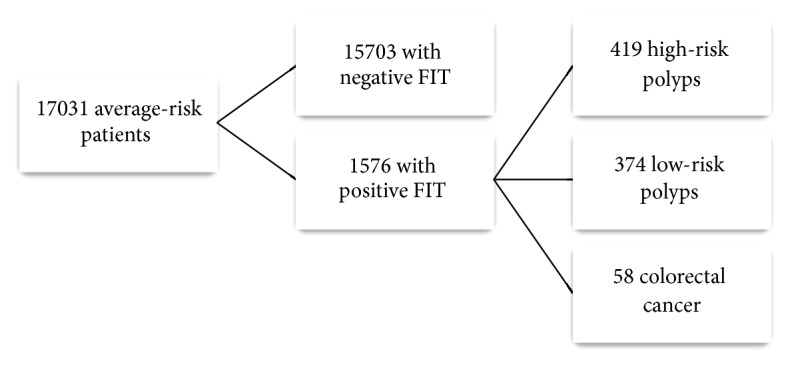
Flow chart demonstrating the study cohort.

From January 2009 to April 2013, 17,031 average-risk men and women 50 to 75 years of age completed at least one round of screening with two FIT kits over the course of the pilot program. The mean age was 63 years (SD: 6), with 57% of participants being male. 1576 (9.3%) had at least one positive FIT and underwent the recommended colonoscopy. Roughly 89% of patients with at least one FIT underwent colonoscopy. Of these, 58 participants (3.7%) had CRC, 419 (31.6%) had high-risk polyps, and 374 (23.7%) had low-risk polyps as their most significant lesion (Figure 1).

The PPV for colorectal neoplasia of two positive FIT results was compared to one positive FIT and shown in Table 1.

## 4. Discussion

Our analysis presents the largest study and first North American data evaluating the effect of multiple FIT specimens per screening round on the detection of colorectal neoplasia. Average-risk participants of a colon screening program had a significantly higher likelihood of CRC or high-risk polyps at colonoscopy, if both FITs were positive. To evaluate the strategy of one FIT per screening round, we compared participants with a positive FIT on the first kit, regardless of the results of the second FIT, to participants whose first FIT was negative and the second FIT was positive. As depicted in Table 2, the second FIT detected CRC or high-risk polyps in additional 105 (6.7%) participants including 7 cases of CRC, accounting for 12% of the total CRC detected in this cohort.

The FIT is an ideal test for population-based screening due to its low cost, low risk, and high acceptance amongst participants. The test performance of FIT may be affected by the prevalence of CRC in the population of interest [20], the site of the lesion within the colon [18], the size of the lesion [21], the FIT brand [22], the time from collection to analysis [23], the interval between screening rounds [24], the cut-off chosen for positivity, and the number of specimens collected per screening round [25].

A recent meta-analysis by Lee et al. [20], evaluating the sensitivity and specificity of FIT for the detection of CRC, found that while the cut-off for positivity affected the FIT performance, the brand and number of specimens per screening round did not. However, the effect of number of specimens on FIT performance did not control for FIT cut-off.

The published literature evaluating the effect of the number of FIT specimens and on the performance of FIT in average-risk, asymptomatic patients is limited and contradictory. In a randomized trial comparing one and two FIT specimens in a Dutch screening population [17], the 2-specimen FIT group had a significantly higher detection rate of advanced neoplasia and a nonsignificant increase in PPV for CRC comparing one positive FIT to both positive FITs in the 2-specimen arm of the trial. Of note, there was no difference in participation between the two groups. Three cohort studies comparing different numbers of FIT specimens performed prior to screening colonoscopy report conflicting results. Nakama et al. [26] and Park et al. [27] demonstrated that two FIT specimens increased the sensitivity for CRC at both the 75 and 100 ng/mL cut-offs.

On the other hand, Hernandez et al. [28] reported that the highest level of 2-specimen FIT strategy versus one FIT sample did not improve the PPV for CRC and was associated with higher cost than one FIT specimen; however, none of the three studies compared the performance of one positive FIT to both positive FITs.

The increased cost of a two-specimen FIT strategy includes the cost of the second FIT and, due to a higher positive rate, the increased demand on colonoscopy and pathology services. Some have suggested that jurisdictions with limited colonoscopy resources perform follow-up colonoscopy after two FIT specimens, only if both tests are positive [17]. In the current study, this would have resulted in 23% of CRC and 50% of high-risk polyps being missed with a 67% decrease in colonoscopies required for that screening round. However, this does not factor in the downstream costs of colonoscopy for eventual cancer diagnosis, advanced cancer treatments for those “missed,” or the downstream savings of early-detected or prevented CRC. In an environment of cost containment, using a single FIT per screening round and selecting a cut-off to control colonoscopy demand are perhaps a better strategy. However, it requires more in-depth study, including cost-effectiveness analysis, which not only assesses the number and frequency of FIT tests, but also evaluates the FIT performance at various cut-offs. In British Columbia, a single quantitative FIT with a lower cut-off (50 ng/mL buffer, 10 micrograms/grams feces) was chosen for our province-wide screening program to decrease the cost of FIT, while maintaining the PPV for neoplasia.

This study has the following limitations. First, colonoscopy, which has a known false negative rate for cancer and adenomas, was used as the gold standard for neoplasia detection. Second, the timeline of the study did not allow for assessing the effect of multiple rounds of screening on the PPV of FIT nor the influence of one versus two FITs on CRC mortality and incidence, the main outcomes of interest for a screening program. Third, as colonoscopies were not performed on participants with two negative FITs, the sensitivity and specificity of FIT cut-off for colorectal neoplasia could not be evaluated. Finally, as the entire cohort completed two FITs, the effect of multiple tests per screening round on participation could not be evaluated. We also included all colonoscopy findings that would lead to a 3-year interval colonoscopy under the definition of high-risk polyps, which may make it more challenging to compare our results with other studies. Our surveillance intervals changed following publication of guidelines recommending that one or two sessile serrated adenomas/polyps less than 10 mm in size, without cytologic dysplasia, undergo surveillance colonoscopy in 5 years [29].

The advantages of our study are the inclusion of a large population of average-risk individuals in the screening age group, novel data on a North American population, and complete data on all participants. To our knowledge, this is the first study to show a significant improvement in the PPV for CRC and high-risk polyps when both FITs are positive compared to one positive FIT in a screening population. All ten Canadian provinces have begun or are planning screening programs, heightening the impact of these results on other jurisdictions.

In conclusion, for average-risk participants of programmatic colon screening, two positive FITs have a higher PPV for the detection of CRC and high-risk polyps than one positive FIT. The added value of the second FIT in our cohort was detection of 12% of the total CRCs and 23% of the total high-risk polyps. Future studies should consider a cost-effectiveness analysis of number of samples per screening round and FIT cut-off on screening population outcomes.

## Figures and Tables

**Table 1:** Positive predictive value of one versus two positive FITs.

Pathology	Two positive FITs	PPV (95% CI)	One positive FIT	PPV (95% CI)	Total	*p* value
Cancer	45	8% (6%, 11%)	13	1% (1%, 2%)	58	<0.0001
High-risk polyp	210	40% (36%, 44%)	209	20% (18%, 22%)	419	<0.0001
Any neoplasia	352	67% (63%, 71%)	499	48% (45%, 51%)	851	<0.0001

**Table 2:** Colorectal cancer and high-risk polyps missed with one FIT specimen.

	1st FIT+	Missed lesions 1st FIT−/2nd FIT+	Total
Cancer only	51 (87.9%)	7 (12.1%)	58
High-risk polyps	321 (65.4%)	98 (23.4%)	419
Any neoplasia	607	244	851
